# Second International Conference on Leprosy

**Published:** 1909-12

**Authors:** 


### Second International Conference on Leprosy.

In the Public Health Reports for September 17, appears the report of Passed Assistant Surgeon Donald H. Currie, of the United States Public Health and Marine Hospital Service, on the Second International Conference on Leprosy held in Bergen, Norway, August 16 to 19, 1909. Dr. Currie was one of the official delegates representing the United States at the conference. In his report, he states that the following enumeration gives the distribution of leprosy throughout the world as obtained from the reports of the official delegates, and from data furnished by the Norwegian government:

CASES A[1]

France, 246; Iceland, 200; Germany, 28; Roumania, 208; Servia, 3; Bulgaria, 9; European Turkey, 550; Greece, 9; Crete, 600; Russia, 1,372; Italy, 123; Spain, 240; Palestine, 800; India, 97,340; Ceylon, 589; Indo-China, 10,500; Java, 15,000; Borneo, 68; Sumatra, 896; Japan, 40,000; Canada, 20; Cuba, 1,297; Jamaica, 115; United States of Colombia, 4,152; Argentine Republic, 12,000; Algeria (in 26 years), 109; United States of America: mainland of America, 146; Hawaiian Islands, 764; Porto Rico, 17; Guam, 19; Philippine Islands, 2,330; Canal Zone, 7.

A brief review of the more important papers read at the conference is given. Dr. Ehlers, of Copenhagen, who recently made a visit to this country, presented the preliminary report of the Danish-French commission for the study of leprosy, the title of his paper being "The Transmission of Leprosy by Suctorial Insects."

The conference adopted the following resolutions:

1. The Second International Scientific Conference on Leprosy confirms in every respect the resolutions adopted by the First International Conference of Berlin, 1897.

Leprosy is a disease which is contagious from person to person, whatever may be the method by which this contagion is effected. Every country, in whatever latitude it is situated, is within the range of possible infection by leprosy, and may, therefore, usefully undertake measures to protect itself.

2. In view of the success obtained in Germany, Iceland, Norway, and Sweden, it is desirable that other countries should isolate lepers.

3. It is desirable that the children of lepers should be separated from their parents as soon as possible, and that they should remain under observation.

4. An examination should be made from time to time of those having lived with lepers by a doctor having special knowledge.

It is desirable that lepers should not engage in certain trades or occupations.

All leper vagabonds and beggars should be strictly isolated.

5. All theories on etiology and the mode of propagation of leprosy should be carefully examined to ascertain if they accord with our knowledge of the nature and biology of the bacillus of leprosy.

6. The clinical study of leprosy induces the belief that it is not incurable. We do not at present possess a certain cure. It is desirable, therefore, to continue the search for a specific remedy with the greatest zeal.

The International Leprosy Conference, held in Berlin in 1897, arrived at the following conclusions: Every leper is a danger to those around him, the danger varying according to the nature and extent of his relations to others and also to the sanitary conditions under which he lives. Among those living in an unsanitary manner, every leper is especially dangerous to his family and fellow-workers. The theory of the heredity of leprosy is becoming less probable, and the contagiousness of the disease is generally accepted. The following resolutions were formally adopted:

1. In countries in which leprosy forms foci or has a great extension, isolation is the best means of preventing the spread of the disease.

2. The system of obligatory notification and of observation and isolation, as carried out in Norway, is recommended to all nations with local self-government and a sufficient number of physicians.

3. It should be left to the legal authorities, after consultation with the medical authorities, to take such measures as are applicable to the special social conditions of the districts.

---

THROUGH the philanthropy of Miss Mary Harriman, eldest daughter of the late E. H. Harriman, the poor children of Brooklyn who are afflicted with tuberculosis are able to keep up with their classes in the public schools and be cured at the same time.

The ferryboat Susquehanna, which Miss Harriman loaned to the Brooklyn Committee on the Prevention of Tuberculosis during the summer as a day camp, reference to which was made in these columns at the time, is being used as a public school this winter for the little consumptives who are not allowed by law to attend the regular schools. The boat is moored at the foot of Columbia street, Brooklyn, in the Erie Basin.

The Board of Education has coöperated with Miss Harriman and the committee, and made the spacious ferryboat an annex of

Public School No. 27. It is fitted with desks, blackboards and all the furniture of the modern schoolroom. Its method of instruction, however, is that of the old-fashioned country school. As there are less than forty pupils from every grade, one teacher must suffice for all. In order to have a teacher who will be sympathetic and not push the children too hard when they are tired, the school has been put in charge of a man who has had consumption, John Birdsell.

Wealthy Brooklyn women are providing milk and eggs for the children during school hours.

---

A GIFT of one million dollars by John D. Rockefeller to fight the "hookworm disease" was announced at the offices of the Standard Oil Company recently. A dozen well-known educators and scientists, in large part from institutions of learning in the south, where the parasite is prevalent, were called in conference with Mr. Rockefeller's representatives at the Standard Oil Offices, at No. 26 Broadway, Tuesday, October 26, 1909, and at that meeting Mr. Rockefeller's desire to organise a commission to carry on a campaign against the malady was discussed. As a result, the Rockefeller Commission for the Eradication of the Hookworm Disease was organised. The members of this commission, as selected by Mr. Rockefeller, are:

Dr. William H. Welch, professor of pathology in Johns Hopkins University, president of the American Medical Association; Dr. Simon Flexner, director of Rockefeller Institute for Medical Research; Dr. Charles W. Stiles, chief of the Division of Zoology, United States Public Health and Marine Hospital Service, and discoverer of the American species of hookworm and the prevalence of the disease in America; Dr. Edwin A. Alderman, president of the University of Virginia; Dr. David F. Houston, chancellor of Washington University, St. Louis; P. P. Claxton, professor of education in the University of Tennessee; J. Y. Joyner, State Superintendent of Education in North Carolina and president of the National Educational Association; Walter H. Page editor of "The World's Work;" Dr. H. B. Frissel, principal Hampton Institute; Frederick T. Gates, one of Mr. Rockefeller's business managers; Starr J. Murphy, Mr. Rockefeller's counsel in benevolent matters, and John D. Rockefeller, Jr.

All but Professor Claxton and Mr. Joyner were present at the meeting Tuesday, and they have both since accepted places on the board.

